# The mediating effect of perceiving close relatives as obese on obesity and weight control behavior score among adults: An exploratory cross-sectional study in Chongqing, China

**DOI:** 10.3389/fpubh.2022.984588

**Published:** 2022-11-07

**Authors:** Tingting Wu, Weiwei Liu, Yifan Chen, Tian Guo, Rong Sun

**Affiliations:** ^1^Chongqing College of Traditional Chinese Medicine, Chongqing, China; ^2^Department of Food and Nutrition, College of Medical and Life Sciences, Silla University, Busan, South Korea; ^3^Research Center for Medicine and Social Development, Chongqing Medical University, Chongqing, China; ^4^School of Public Health, Research Center for Public Health Security, Chongqing Medical University, Chongqing, China; ^5^School of Public Health, Cheeloo College of Medicine, Centre for Health Management and Policy Research, Shandong University, Jinan, China; ^6^Chongqing Health Education Institute, Chongqing, China; ^7^Department of Physical Examination, The First Affiliated Hospital of Chongqing Medical University, Chongqing, China

**Keywords:** obesity, mediating effects, perceived obesity, close relatives, health behavior

## Abstract

Obesity is one of the most glaringly obvious, yet most neglected, public health problems the world is facing today. Individuals' self-perception of being overweight is essential to engaging in weight control behavior. This is the first exploratory study in Chongqing to assess the mediating effect of perceived obesity in close relatives on obesity and weight control behavior among adults. A cross-sectional study, including 3,7492 participants, was conducted in a healthcare center in Chongqing, China. For Kruskal–Wallis test analyses, logistic regression and Sobel-Goodman mediation tests were employed. Only 1.76% of participants (660) were medically identified as obese, and only 2.13% of participants (798) thought their close relatives were obese. Nearly one-third of the participants consumed more than double the daily recommended amount of meat. More than 85% of participants were physically inactive. Obesity was positively associated with obesity perception in close relatives (OR = 19.556, *P* < 0.001). The association between the respondent's obesity status and weight control behavior scores changed statistically significantly (β = 0.594, *P* < 0.001). Individuals who perceived their close relatives as obese were more likely to engage in weight control behavior themselves (β = 0.678, *P* = 0.001). The obesity perception in close relatives partially mediated the association between obesity and weight control behavior (ab = 0.141, SE = 0.03, 95% CI = 0.086, 0.198). Obese people are more likely to engage in weight control behaviors. The effect of individuals' obesity status on weight control behavior scores is partially mediated by the obesity perception in close relatives among the participants. Findings suggest that personal obesity status perceptions of close relatives might provide new weight management ideas for healthcare centers.

## Introduction

The prevalence of obesity over the last 30 years has increased steadily worldwide. From 1999 to March 2020, the prevalence of obesity in the US increased from 30.5 to 41.9% (1). The prevalence of overweight and obesity among Australians aged 18 and over increased from 57% in 1995 to 67% in 2017–2018 (2). Nearly two-thirds of adults in Europe are either overweight or obese, which has reached “epidemic” proportions across Europe and is still rising, reported by the WHO Office for Europe said in a published study (3). Unfortunately, despite China's status as a developing country, the prevalence estimates for overweight and obesity in adults for the period 2015–19 are 34.3% for overweight and 16.4% for obesity (≥18 years) (4). Researchers pointed out that, if current trends continue, global obesity prevalence will reach 18% to 21%, and severe obesity will surpass 6–9% by 2025 (5).

As one of the most glaringly visible, ye most neglected, public health problems the world is facing, obesity strains individuals and families, affecting overall health, healthcare costs, and productivity. The escalating obesity pandemic is associated with cardiovascular disease, type-2 diabetes, obesity-related cancers, osteoarthritis, and psychological disturbance, which account for much of the mortality and years of life lost (6–8). People with obesity experience a four-fold increased risk of developing severe COVID-19 (9, 10). The considerable direct and indirect costs put pressure on the healthcare system. People who are obese have a 30% higher medical cost than those with a normal body mass index (BMI). Total healthcare expenses associated with treating obesity and related conditions double every decade, presenting a significant financial burden for patients (11).

Previous studies reported that the self-perception of being overweight is crucial for people with obesity to understand so as to initiate weight control practices and gain the desire to reduce weight (12). Weight misperception among individuals with obesity might hinder the successful implementation of obesity prevention and management interventions (13, 14). Thus, the use of perception-improving approaches in public health and clinical practice has been advocated (13–16). Several theoretical models of health behavior presented perception as an essential individual-level concept in explaining behavior and choices (17). As described in the Health Belief Model (18), when individuals perceive a condition as severe or significantly risky, they are more likely to become active to counteract it. According to the theory of planned behavior (19), the individual's perception of social pressure regarding a condition is a strong predictor of the person's actions, and they are more likely to act on their behavioral intentions when their perceived behavioral control is high.

Most research focused on the role of perceptions of their body weight that patients who are overweight or obese have in their weight control behavior and disease development. Studies discovered that when people with obesity acknowledge that they are overweight, they are more likely to engage in weight control behaviors (12, 20). In addition, some systemic reviews suggested that the self-perception of obesity may also mediate the consequences of obesity through health behaviors (21, 22). However, perception likely plays a role in every aspect of life, such as family and society.

Many studies indicated that, in China, a country with a strong family-oriented culture, family factors may significantly impact individual weight control behaviors and health outcomes. Parents' perceptions of their children's weight or obesity and personal perceptions of their weight are often discussed (14, 23–25), while few studies investigated perceptions of obesity in close relatives. Therefore, this study aimed to investigate individuals' weight control behaviors and their perceptions of obesity in close relatives, including whether there is a mediating effect of individuals' perceptions of obesity in close relatives on the relationship between their weight control behaviors and the prior occurrence of obesity, which could contribute to more appropriate weight management strategies.

## Method

### Participants

Data were obtained from a health center located in a medical university-affiliated hospital in Chongqing, China. A total of 38,756 individuals underwent health checkups from July 2020 to January 2022 at the center. Individuals who met the following criteria were included in the analysis: (1) age of 18 years and above and living in Chongqing for at least 6 months; (2) voluntarily signed the informed consent form; and (3) no metabolic diseases, such as congenital obesity. Finally, a total of 37,492 participants met the criteria to include data analysis.

### Measurements

All participants were invited to complete a questionnaire through a website that gathered information on demographic characteristics (age, gender), perceived obesity in close relatives, participants' obesity status, and health-related habits. The health-related habits were designed based on the Health Promotion Lifestyle Scale and the Chinese Dietary Guidelines (2016 version) (26, 27), and the three aspects of personal diet and dietary intake behavior, exercise behavior, and smoking and alcohol consumption behaviors were included in the data analysis regarding weight control.

### Obesity

As detected by a doctor, participants with BMI ≥ 27.5 kg/m were considered obese. Obesity refers to BMI ≥ 27.5 kg/m according to the World Health Organization recommendations for the Chinese population (28).

### Weight control behavior

The fundamental cause of obesity and overweight is an energy imbalance between calories consumed and calories expended (29). When individuals are obese or a family member becomes obese, they may regulate their weight or lose weight through behaviors such as dieting and exercising. Thus, the weight control behaviors included dietary behaviors and exercise behaviors. Dietary behaviors included an average daily intake of various food groups in the last month, average daily water intake, frequency of skipping breakfast, late-night snacks per week, and poor dietary habits. The average daily intake of food groups included rice and noodles, meat, fish, vegetables, and fruits. Measurement was based on the Chinese Dietary Guidelines. Poor dietary habits included drinking water with meals, eating too fast, overeating, and eating late (dinner). Exercise behavior included the average daily sedentary time, average weekly exercise time, and exercise intensity in the last month.

The weight control behavior score was measured by assigning a value for dietary and exercise behaviors based on the recommendations of the Chinese Dietary Guidelines. The guidelines recommend specific daily amounts of various types of food and water specifically for adults and the appropriate amount and duration of daily exercise. If the behavior reaches the recommended standards of the Chinese Dietary Guidelines, 1 point is scored; otherwise, zero points (0) are scored. Higher weight control behavior scores indicate better weight control behavior of the participants.

### Perceived obesity in close relatives

Perceived obesity in close relatives refers to participants' subjective perceptions of whether their close relatives were obese. Moreover, it is unknown whether their close relatives were obese. The participants were asked to answer five questions: “do you think your ______ is obese?” The five genres of family members were father, mother, grandparents, maternal grandparents, and siblings. The questions were close-ended.

### Statistics analysis

Excel 2016 was used to double-check and clean all web questionnaire data. A total of 37,492 participants were finally included in the data analysis. All the data were analyzed with Stata statistical software (version 17.0; College Station, TX 77845, USA). First, dietary and exercise behaviors used frequencies (percentage). Second, the comparisons across different groups were analyzed using the Kruskal–Wallis (K–W) test. Third, logistic regression was employed to explore the association between obesity and perceived obesity in close relatives. Fourth, to test the mediation effect of perceived obesity in close relatives on the association between health behavior and obesity, we conducted the following analysis based on the technique proposed by MacKinnon (30): (1) to estimate the association between obesity and the weight control behavior score using linear regression and (2) to explore further the relationship between obesity and the weight control behavior score when perceived obesity in close relatives was included. All statistical analyses set a statistically significant P-value of < 0.05. The nonparametric bootstrapping technique was used to assess the total, indirect, and direct effects of the mediation model based on 5000 bootstrap samples. If 95% of the confidence intervals (Cis) excluded zero, the effects indicated significant levels. The results include odds ratios (OR), coefficients (β), and 95% confidence intervals CIs.

## Results

Although less than one-third of the participants had a history of smoking, more than half had a history of consuming alcohol. According to the Chinese Dietary Guidelines, nearly one-third of the participants consumed more than two times the daily recommended amount of meat, while fewer than one-third of the participants consumed less than one-third of the recommended daily intake of fish. Nearly half of the participants had late-night snacks almost every day and drank less than 1,200 ml of water. More than 85% of participants were physically inactive; their exercise time was less than 60 minutes a week. Only 1.76% of participants (660) were medically diagnosed to be obese. The behavioral characteristics of those who are obese and those who are not obese are shown in Table 1. People who are obese and people who are not obese did not behave similarly in any way except for their daily rice and noodle consumption, daily fruit consumption, frequency of missing breakfast, and intensity of exercise (*P* < 0.05).

**Table 1:** Health behavior characteristics of the obese and non-obese population in Chongqing (*N* = 37,492).

**Characteristics**	**subgroups**	**Normal (*n =* 36,832)**	**Obese (*n =* 660)**	**Total**	***P*-value**
Age		42.50 ± 11.74	47.35 ± 10.95	42.58 ± 11.74	<0.001^*^
Gender	Men	17,745 (48.18%)	504 (76.36%)	18,249 (48.67%)	<0.001^*^
	Women	19,087 (51.82%)	156 (23.64%)	19,243 (51.33%)	
Alcohol drinking status	Never-drinker	2,448 (6.65%)	106 (16.06%)	2,554 (6.81%)	<0.001^*^
	Current drinker	34,111 (92.61%)	542 (82.12%)	34,653 (92.43%)	
	Former drinker	273 (0.74%)	12 (1.82%)	285 (0.76%)	
Smoking status	Never smoker	4,700 (12.76%)	125 (18.94%)	4,825 (12.87%)	<0.001^*^
	Current smoker	31,092 (84.42%)	494 (74.85%)	31,586 (84.25%)	
	Former smoker	1,040 (2.82%)	41 (6.21%)	1,081 (2.88%)	
Second smoking	Never	1,844 (5.01%)	44 (6.67%)	1,888 (5.04%)	0.003^*^
	Sometimes	1,421 (3.86%)	28 (4.24%)	1,449 (3.86%)	
	Often	3,246 (8.81%)	80 (12.12%)	3,326 (8.87%)	
	Always	30,321 (82.32%)	508 (76.97%)	30,829 (82.23%)	
Average daily intake of rice and noodles	<200 g	29,575 (80.30%)	532 (80.61%)	30,107 (80.30%)	0.93
	200 g~ <400 g	6,454 (17.52%)	115 (17.42%)	6,569 (17.52%)	
	≥400 g	803 (2.18%)	13 (1.97%)	816 (2.18%)	
Average daily intake of meat	<50 g	8,062 (21.89%)	104 (15.76%)	8,166 (21.78%)	<0.001^*^
	50g~ <100 g	19,821 (53.81%)	303 (45.91%)	20,124 (53.68%)	
	>100g	8,949 (24.30%)	253 (38.33%)	9,202 (24.54%)	
Average daily intake of fish	<50g	23,429 (63.61%)	378 (57.27%)	23,807 (63.50%)	<0.001^*^
	50g~ <100g	10,944 (29.71%)	217 (32.88%)	11,161 (29.77%)	
	≥100g	2,459 (6.68%)	65 (9.85%)	2,524 (6.73%)	
Average daily intake of vegetables	<300g	28,242 (76.68%)	472 (71.52%)	28,714 (76.59%)	0.005^*^
	300g~ <500g	6,572 (17.84%)	139 (21.06%)	6,711 (17.90%)	
	≥500g	2,018 (5.48%)	49 (7.42%)	2,067 (5.51%)	
Average daily intake of fruit	<200g	29,879 (81.12%)	527 (79.85%)	30,406 (81.10%)	0.67
	200g~ <400g	5,563 (15.10%)	108 (16.36%)	5,671 (15.13%)	
	≥400g	1,390 (3.77%)	25 (3.79%)	1,415 (3.77%)	
The frequency of breakfast skipping	Always (7 days/week)	655 (1.78%)	14 (2.12%)	669 (1.78%)	0.087
	Often (3–6 days/week)	1,473 (4.00%)	15 (2.27%)	1,488 (3.97%)	
	Sometimes(1~2days/week)	11,197 (30.40%)	191 (28.94%)	11,388 (30.37%)	
	Never	23,507 (63.82%)	440 (66.67%)	23,947 (63.87%)	
The frequency of midnight snack eating	Never	5,565 (15.11%)	124 (18.79%)	5,689 (15.17%)	0.007^*^
	Sometimes(1~2days/week)	1,595 (4.33%)	35 (5.30%)	1,630 (4.35%)	
	Often (3–6 days/week)	11,308 (30.70%)	171 (25.91%)	11,479 (30.62%)	
	Always (7 days/week)	18,364 (49.86%)	330 (50.00%)	18,694 (49.86%)	
Intensity of exercise	Light	26,020 (70.65%)	462 (70.00%)	26,482 (70.63%)	0.70
	Moderate	10,183 (27.65%)	189 (28.64%)	10,372 (27.66%)	
	Heavy	629 (1.71%)	9 (1.36%)	638 (1.70%)	
Accident Suffered	Yes	728 (1.98%)	24 (3.64%)	752 (2.01%)	0.003^*^
	No	36,104 (98.02%)	636 (96.36%)	36,740 (97.99%)	
Adequate sleep	Yes	8,556 (23.23%)	177 (26.82%)	8,733 (23.29%)	0.031^*^
	No	28,276 (76.77%)	483 (73.18%)	28,759 (76.71%)	
Mental stress	None	1,010 (2.74%)	39 (5.91%)	1,049 (2.80%)	<0.001^*^
	Mild	4,848 (13.16%)	117 (17.73%)	4,965 (13.24%)	
	Severe or worse	30,974 (84.10%)	504 (76.36%)	31,478 (83.96%)	

* P < 0.05.

Only 2.13% of participants (798) thought their close relatives were obese. After adjusting for other health behavior variables, obesity was positively associated with obesity perception in close relatives (OR = 19.556, *P* < 0.001), which showed that adults with obesity were more likely to think their relatives were obese than those without obesity (Table 2). The participants who had suffered an accident, such as a relative passing away last year, were more likely to perceive that their close relatives were obese than those who had never suffered an accident (OR = 0.617, *P* < 0.05). Those who had adequate sleep were more likely to report overweight relatives (OR = 0.822, *P* < 0.05).

**Table 2:** Factors associated with obesity perception in close relatives among adults in Chongqing, China.

**Variables**	**Subgroup**	**OR**	**95% CI**	***P*-value**
Obesity status of respondents	Normal	Ref.	Ref.	
	Obese	19.556	15.992,23.912	< 0.001^*^
Age		1.006	1.033–1.012	0.049^*^
Gender	Men	Ref.	Ref.	
	Women	1.060	0.902–1.245	0.480
Alcohol drinking status	Never-drinker	Ref.	Ref.	
	Current drinker	0.948	0.716–1.255	0.710
	Former drinker	0.632	0.243–1.649	0.349
Smoking status	Never smoker	Ref.	Ref.	
	Current smoker	1.253	0.973–1.612	0.080
	Former smoker	1.101	0.687–1.762	0.690
Second smoking	Never	Ref.	Ref.	
	Sometimes	0.842	0.501–1.415	0.516
	Often	0.911	0.603–1.376	0.657
	Always	1.067	0.758–1.502	0.712
Accident Suffered	Yes	Ref.	Ref.	
	No	0.617	0.416–0.915	0.016^*^
Adequate sleep	Yes	Ref.	Ref.	Ref.
	No	0.822	0.693–0.974	0.024^*^
Mental stress	None	Ref.	Ref.	
	Mild	1.281	0.840–1.952	0.250
	Severe or worse	0.920	0.614–1.379	0.688

* P < 0.05.

**Figure 1:**
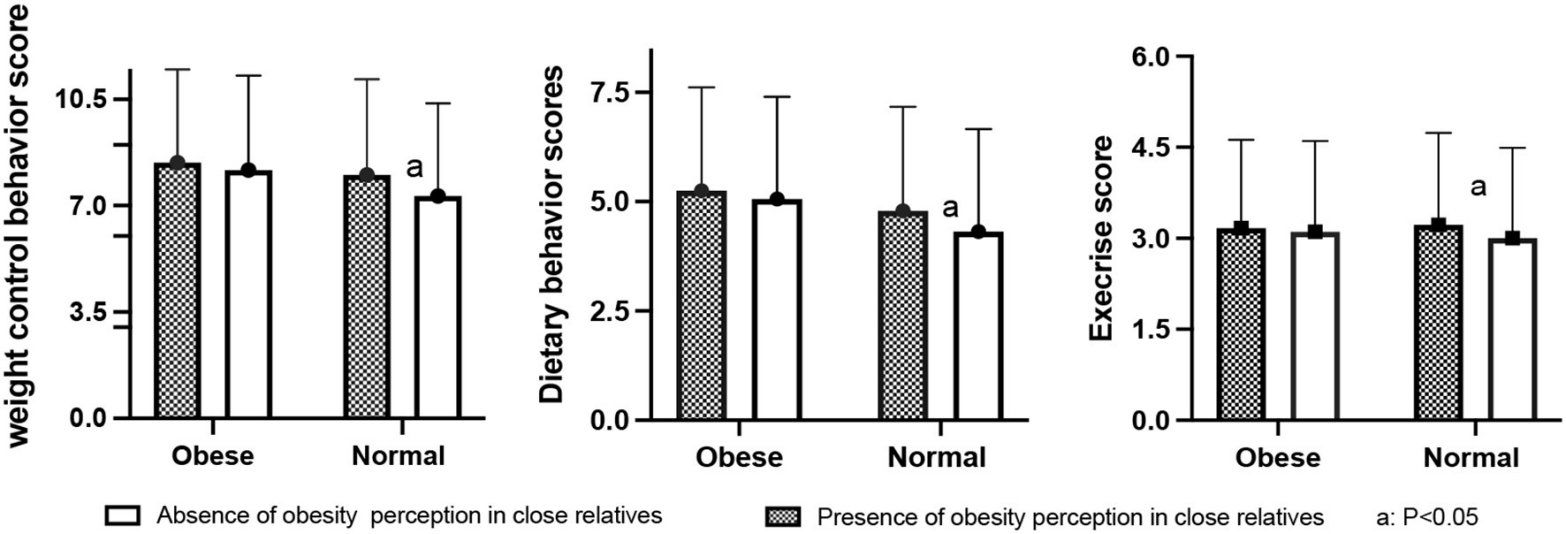
Shows the weight control score with different perceptions of close relatives.

Figure 1 shows that individuals with obesity had higher weight control behavior scores than individuals who are not obese (8.23 ± 3.10 vs. 7.33 ± 3.05, *P* < 0.01, Kruskal–Wallis test). Among the normal individuals, those who perceived their close relatives as obese had higher weight control behavior scores compared to those whose closest relatives were not obese (*P* < 0.05), and they also had significantly higher dietary and exercise scores (*P* < 0.05). The same results were found for individuals who are obese, except with insignificant differences. As shown in Table 3, the model without mediators (obesity perception in close relatives) indicated that the obesity status of the respondent was correlated with weight control behavior scores (β = 0.819, *P* < 0.001).

**Table 3:** The mediating effect of obesity perception in close relatives on the association between obesity and weight control behavior scores among adults in Chongqing, China.

		**Model without mediators**	**Model with mediators**
**Variables**	**Subgroup**	**β (95%CI)**	**β (95%CI)**
Obesity status of respondents	Normal	Ref.	Ref.
	Obese	0.819(0.495, 0.965)^**^	0.594(0.347, 0.828)^**^
Obesity Perception in close relatives	Normal	/	Ref.
	Obese	/	0.678(0.380, 0.816)^**^
Age		0.018(0.015, 0.023)^**^	0.017(0.015, 0.021)^**^
Gender	Men	Ref.	Ref.
	Women	−0.370(−0.438, −0.302)^**^	−0.370(−0.438, −0.302)^**^
Alcohol drinking status	Never-drinker	Ref.	Ref.
	Current drinker	−0.043(−0.017, 0.085)	−0.043(−0.017, 0.085)
	Former drinker	0.453(0.079, 0.827)^*^	0.459(0.084, 0.832)^*^
Smoking status	Never smoker	Ref.	Ref.
	Current smoker	0.613(0.509, 0.717)^**^	0.611(0.507, 0.714)^**^
	Former smoker	0.750(0.547, 0.953)^**^	0.750(0.546, 0.952)^**^
Second smoking	Never	Ref.	Ref.
	Sometimes	−0.2557(−0.464, −0.048)^*^	−0.254(−0.464, −0.048)^*^
	Often	0.068(−0.105, 0.240)	0.068(−0.105, 0.240)
	Always	0.09(−0.05, 0.235)	0.084(−0.055, 0.234)
Accident Suffered	Yes	Ref.	Ref.
	No	−0.086(−0.306, 0.134)	−0.077(−0.297, 0.142)
Adequate sleep	No	Ref.	
	Yes	0.081(0.005, 0.158)^*^	0.0839(0.008, 0.160)^*^
Mental stress	None	Ref.	Ref.
	Mild	−0.069(−0.272, 0.134)	−0.072(−0.275, 0.131)
	Severe or worse	−0.0210(−0.400, −0.0180)^*^	−0.0207(−0.398, −0.017)^*^

* P < 0.05,

** P < 0.001.

When adding the obesity perception for close relatives into the model (Table 3), the association between the respondent's obesity status and weight control behavior scores showed a statistically significant change (β = 0.594, *P* < 0.001). After controlling for other health behaviors, the adults who perceived their close relatives as obese were more like engage in weight control behavior (β = 0.678, *P* = 0.001). Bootstrapping was used to further verify the mediation effect, and statistically significant direct, indirect, and total effects were found. The obesity perception of close relatives had a partial mediating effect in the association between obesity and weight control behavior (ab = 0.141, SE = 0.03, 95% CI = 0.086, 0.198). The mediating effect can explain 17% of the total effect of obesity on weight control behavior scores (Table 4).

**Table 4:** Total, direct and indirect effects of obesity on weight control behavior scores (WCDS).

**Item**	**β**	**SE**	**95%CI**
Total effects of obesity on WCDS	0.819	0.12	0.495, 0.965
Indirect effects of obesity on WCDS	0.141	0.03	0.086, 0.198
Direct effects of obesity on WCDS	0.678	0.13	0.428, 0.917

## Discussion

Chongqing, located in the southwest, had the largest resident population in China in 2020, according to the seventh national population census in China (31). This study found that only 1.7% of the participants were diagnosed as obese by doctors. However, a previous study showed that the burden of obesity is severe in Chongqing (32). This may be because this survey was conducted at a healthcare center where participants might pursue weight loss under the healthcare center's management. We found that the participants' diet and exercise behaviors were unhealthy. Most of the participants' behaviors deviated from the Chinese Dietary Guidelines. Following an unhealthy diet and the lack of exercise are consistent with the results of previous nutrition surveys reported in China (4). We found that individuals who are obese were more likely to perceive their close relatives as obese than individuals who are nont obese, after adjusting for other health behavior variables. One possible reason is that as the overall obesity trend in society rises, individuals' perceptions of obesity in close relatives may increase with the increased personal obesity risk and the dissemination of obesity information. Individuals with obesity already experiencing the abovementioned problem might be more concerned about the obesity status of close relatives.

We found that respondents with obesity were more likely to produce higher weight control behavior scores, which is consistent with previous studies (33–35). Individuals diagnosed as obese appear more motivated to manage their weight, as self-identification as overweight is associated with greater self-reported weight loss intentions and/or attempts to lose weight (35). Conversely, a higher prevalence of unhealthy weight control behaviors was evident among overweight adolescents (36). Unlike adolescents, adults already have a certain stock of health knowledge and a more mature understanding of their health status. They are the ones responsible for their health. When individuals are diagnosed with obesity, they may have already experienced emotional distress, be aware of the crisis following treatment or advice from a doctor, and seek change and dignity through weight loss (37).

In addition, we found that adults who perceived their relatives as obese had a higher score for healthy weight control behavior, which indicated that perceiving close relatives as obese may be crucial for individuals engaging in weight control behavior after perceiving themselves as obese (12). The Health Belief Model posits that perceiving obesity as a risk factor for other diseases is crucial to changing health behavior (18). Perceiving close relatives as obese may increase the sense of crisis. When an individual perceives a condition as severe or as a severe risk, they are more likely to act to counteract the condition. We also found that, by perceiving close relatives as obese, the effect of an individual's obesity status on weight control behavior scores becomes weaker. This result suggests that perceiving close relatives as obese, to some extent, mediates the effect of an individual's obesity on weight control behavior. Individuals with obesity were more likely to perceive their close relatives as obese, which might encourage them to exercise weight control. When intervening in obesity prevention, variables other than one's own weight perception should be considered when close relatives are perceived to be obese. While perceiving close relatives as obese, factors beyond personal weight perception when intervening in obesity prevention should be considered.

In addition, studies on self-weight perception also found that perceived overweight or obesity may lead to numerous unhealthy conditions and are associated with future weight gain (22, 38, 39). An obesity self-weight perception may cause obesity anxiety, which is associated with obesity and may increase individual stress, leading to future eating and physiological disorders (40). Thus, misperceptions of obesity in close relatives may cause similar consequences. The next step of our study is to further explore the accuracy of perceptions of obesity in close relatives.

The present study has several limitations. First, it was based on the analysis of cross-sectional data; the causal relationship between perceiving obesity in close relatives and weight control behavior could not be determined. Further longitudinal studies are required. Second, this study was only conducted for the adult population in one Chongqing health care center; additional studies should be conducted in different communities and multiple regions to confirm the reliability of the findings. Third, sociodemographic information such as income and education level were not collected because of time constraints and respondents' privacy concerns. Thus, the proportion of the mediating effect of perceived obesity in close relatives on the total effect was relatively low, and exploration of additional mechanisms related to obesity and weight control behaviors is required.

Despite these limitations, the present study is the first exploratory study in Chongqing to assess the mediating effect of perceived obesity in close relatives on obesity status and weight control behavior in adults. Family clustering of obesity exists, reflecting the effects of shared genetics and environment among close relatives (41). Adults are sensitive to their weight. When family members are similar in body size or the family as a whole is obese, it may cause a perceived reduced threat of obesity (42). Several studies showed that individuals' obesity self-perception significantly impacts weight control behavior and obesity outcomes (12, 23, 25). The results suggest that individuals' perceptions of obesity in close relatives might also play a significant role in management interventions using cognitive-related theories, which may further promote behavior change.

## Conclusion

This exploratory study found that individuals who are obese are more likely to engage in weight control behaviors. The effect of individuals' obesity status on weight control behavior scores is partially mediated by perceived obesity in close relatives among adults visiting a healthcare center in Chongqing, China. In addition, there is an association between individuals' perceptions of close relatives' obesity and weight control behavior. Our findings may further increase behavior change. Future studies should not only focus on an individual's self-perception but also consider the perception of family members, which may provide new weight management ideas for healthcare centers.

## Data availability statement

The original contributions presented in the study are included in the article/supplementary material, further inquiries can be directed to the corresponding author.

## Ethics statement

The studies involving human participants were reviewed and approved by the Ethics Committee of the First Affiliated Hospital of Chongqing Medical University (2020426). The patients/participants provided their written informed consent to participate in this study.

## Author contributions

TW and WL acquired and interpreted data and drafted the manuscript. YC and interpreted the data. TG helped revised the manuscript. RS made significant contributions to the concept, design, participated in the analysis and interpretation of the data, revised the manuscript, and finally approved the version to be published.

## Funding

This study was supported by the Chongqing Education Science Planning Project and Curriculum Research on Emergency Prevention and Control of Epidemic Risk in Medical Colleges and Universities (2020-YQ-06) and Chongqing Science and Health Joint Medical Research Project (Grant Number 2022MSXM023).

## Conflict of interest

The authors declare that the research was conducted in the absence of any commercial or financial relationships that could be construed as a potential conflict of interest.

## Publisher's note

All claims expressed in this article are solely those of the authors and do not necessarily represent those of their affiliated organizations, or those of the publisher, the editors and the reviewers. Any product that may be evaluated in this article, or claim that may be made by its manufacturer, is not guaranteed or endorsed by the publisher.
